# Gregory’s Outlines of Chemistry

**Published:** 1851-11

**Authors:** 


*Gregory's Outlines of Chemistry.* Cincinnati: Derby & Co. 1851.

This treatise on chemistry, by Prof. Gregory, has two peculiarities. In the first place the usual introductory chapters on heat, light, and electricity, are altogether omitted: and in the second place above one half of the volume is occupied with organic chemistry: giving therefore to the latter subject a greater prominence than is usually accorded to it in elementary books. This portion of the work is filled, in large part, with descriptions of new compounds, which the labors of the last few years have brought forth to our knowledge; and with new views and hypotheses founded thereon. Probably any fault that one might be disposed to find with the arrangement and proportionate prominence given to the various items under this head would apply, in a greater or less degree, to all books on chemistry written for students. Entire accordance of views is not indeed to be expected in any department of science. A just and proportionate development of this subject is rendered doubly difficult by the reason of its being in such a rapid transition state that the most perfect and well balanced account of the facts and doctrines of to-day may become somewhat out of date, and obsolete to-morrow. The book, however, we think a good one, and will for a time, till the rapid progress of the science shall make it obsolete, fill its place among its myriad text-books. In its verbal style it is neat, clear, and distinct.

It may be a small matter, but we are disposed to take exception to the expression on the title-page, "revised, corrected and enlarged," by the American editor; as implying that the book has undergone material alterations in his hands. An editor pleases our taste best, who suffers an author to convey his own ideas in his own way. But "de gustibus, &c.," and others may prefer the opposite. We believe, however, in the present case, that the revisions have not modified it essentially, and that the American edition is still, to all intents and purposes, the work of the English chemist.

In its mechanical execution the book is neatly — we might say elegantly got up by the enterprising publishers, Derby & Co., of Cincinnati. It forms a large 12 mo. volume of about 600 pages; and last, not least, possesses a good table of contents and a copious index. G. H.

For sale by Geo. H. Derby & Co., Buffalo, N. Y.

